# Nephroblastoma overexpressed protein (NOV) enhances 5-Fu-mediated inhibitory effect of colorectal cancer cell proliferation via JNK/AP-1/caspase-8/caspase-3 pathway

**DOI:** 10.1007/s12672-021-00403-y

**Published:** 2021-03-22

**Authors:** Dong Wang, Tingting Wang, Yongbo An, Lan Jin, Jin Wang, Guocong Wu, Hongwei Yao, Zhongtao Zhang, Jun Li

**Affiliations:** Department of General Surgery, Beijing Friendship Hospital, Capital Medical University; Beijing Key Laboratory of Cancer Invasion and Metastasis Research & National Clinical Research Center for Digestive Diseases, 95 Yong-an Road, Xi-Cheng District, Beijing, 100050 China

**Keywords:** Nephroblastoma overexpressed protein, Colorectal cancer, 5-fluorouracil, Nude mouse

## Abstract

Chemoresistance often occurs during 5-fluorouracil (5-Fu) treatment of colorectal cancer (CRC). It is significant to explore the potential strategies to sensitize colorectal cancer cells to 5-Fu treatment. We studied the sensitization of Nephroblastoma overexpressed protein (NOV) on 5-Fu treatment. NOV was overexpressed and knocked down in HT115 and RKO cells respectively. Cell proliferation experiments and related mechanism studies by RT-qPCR and Western blot were performed Subsequently. Nude mouse xenograft model was established to test the inhibitory effect of 5-FU on CRC cells in vivo. In this study, we found that NOV mRNA expression was significantly lower in tumor tissues than that in the normal tissues (P < 0.05). The cell proliferation was reduced in the HT115-NOVexp groups (P < 0.05) and increased in the RKO-NOVkd groups (P < 0.05) than that in the control groups and NC groups. The RT-PCR and Western Blot results showed that NOV inhibited the expression of activator protein (AP)-1 (P < 0.05) and promoted the expression of Caspase-8/3 (P < 0.05) in CRC cells in vitro. NOV also improved the inhibitory effect of 5-Fu on inhibiting colorectal cancer proliferation in a tumor cell xenotransplantation nude mouse model. NOV inhibited the expression of AP-1 and JUK and promoted the expression of Caspase-8/3 in cancer tissues in a tumor cell xenotransplantation nude mouse model. In summary, NOV can sensitize CRC cells towards 5-Fu-mediated inhibitory effect on cell proliferation and its sensitization may be achieved by the JNK/AP-1/Caspase-8/Caspase-3 pathway.

## Introduction

The incidence and mortality of colorectal cancer (CRC) are increasing every year, greatly threatening the health of humans [1]. CRC ranked third in men and second in women of the new cases of malignant tumors worldwide [2, 3]. This trend is similar in China, with CRC ranked as fourth and fifth most common cancer among all cancer types in women and men, respectively [4]. And CRC imposes a severe burden on the family of patients and society [5].

Over the past decade, the comprehensive treatment of CRC has been gradually promoted, which results in an improved prognosis [5]. However, more than half of the patients with CRC cannot benefit from the current first-line treatment program. Currently, the 5-fluorouracil (5-Fu)-based strategy is a first-line chemotherapy for CRC [6, 7]. 5-FU exerts anticancer effects by inhibiting tumor cell proliferation and promoting apoptosis [8]. However, the effective rate of the single 5-FU is only about 10–20% [9]. Therefore, it is of great significance to explore the potential strategies to sensitize 5-Fu treatment and provide experimental evidence for finding new therapeutic targets.

Nephroblastoma overexpressed protein (NOV, also called CCN 3), an extracellular matrix protein, belongs to the CCN protein family [10]. NOV plays important biological functions in regulating cell apoptosis, tumor invasion, and tumor metastasis, etc. [11]. Some studies have shown that NOV exerts an inhibitory function in some malignant solid tumors [13–16]. Gupta et al. overexpressed NOV in glioma cells and found that overexpressed NOV inhibited the growth of glioma cells [12]. Fukunaga-Kalabis et al. found that NOV inhibited the invasion of melanoma cells by inhibiting matrix metalloproteinase-2/-9 ( MMP-2/-9), which suggested that low expression of NOV might be a possible mechanism for melanoma progression [13]. NOV expression was reduced in malignant adrenal tumors but unchanged in benign adrenal tumors, which suggested that a low expression of NOV is related to the occurrence of adrenal malignant tumors [14]. In contrast to NOV, abnormal activation of activator protein 1 (AP-1) related pathway is considered to be related to the occurrence of CRC [15, 16]. As previously reported by our team, low NOV expression correlates with disease progression in CRC [17]. Therefore, we focused on the sensitization of NOV on 5-Fu treatment of CRC by in vitro and in vivo experiments.

## Materials and methods

### Cell culture

Two human colon cancer cell lines (RKO and HT115) were bought from ATCC (American Type Culture Collection, USA). The cells were cultured with a DMEM medium at 37 °C, 5% CO_2_.

### Human colorectal cancer tissues

Ten CRC tumor specimens and adjacent normal colorectal tissue were collected during surgery. The protocol was approved by the Ethical Committee of Beijing Friendship Hospital and the informed consent forms were obtained from all patients.

### Reverse Transcription-Polymerase Chain Reaction (RT-PCR)

Total RNA was extracted from the CRC and adjacent normal tissues and CRC cells. The reverse transcription of RNA into cDNA was performed according to the manufactory guidelines (Genecopoeia corp.). 1 μl of 2.5 U/μl polyA polymerase, 1 μl of RTase Mix, and 5 μl of 5 × Reaction Buffer were added into 18 μl of RNA extraction solution. The reaction was proceeded at 37 °C for 60 min, then at 85 °C for 5 min. 10 μl of the reverse-transcribed cDNA was diluted at a ratio of 1:10 to prepare for the next real-time PCR reaction. 10 μl of 2 × all-in-one qPCR Mix, 2 μl of all-in-one qPCR primer (2 μM), 2 μl of universal adaptor PCR primer (2 μM), 5.6 μl of diluted cDNA products, and 0.4 μl of 50 × ROX Reference Dye were mixed together. Then the quantitative real-time PCR was carried out. The primers used were shown in Table 1. The experiments were repeated three times.

**Table 1:** The primers used for PCR

Gene name	Species	Forward primers (5′-3′)	Reverse primer (5′-3′)
NOV	human	CTGTGAACAAGAGCCAGAG	ACTGAACCTGACCGTACACT TGAACTGCAGGTGGAT
AP-1	human	CCTGAGTCTCACTGAGCGTCTGTAC	GTACAGACGCTCAGTGAGAC TCAGG
Caspase 3	human	GGCGTGTCATAAAATACCAG	ACTGAACCTGACCGTACAACA AAGCGACTGGATGAA
Caspase 8	human	AAGCCCAAGCTCTTTTTC	ACTGAACCTGACCGTACAGTTACTGCCAGGGGACTC

*NOV* Nephroblastoma overexpressed protein, *AP-1* activator protein-1

### Cell viability assay

HT115 cells (2 × 10^5^), seeded in 6-well plates, were treated with various concentrations of 5-Fu for 48 h. The cytotoxic effect of 5-FU on HT115 cells was detected by crystal violet staining and the IC50 was calculated.

### Stable transfection of cancer cells

The cells were digested with cell digestion solution. 2 ml of DMEM medium was added for cell suspension. 400 μl of the cell suspension was taken into electric rotating cups. Subsequently, 1 μg of NOV overexpressed or NOV empty plasmids were added in the HT-115 medium, and 1 μg of silenced plasmids and NOV empty plasmids were added in the RKO medium respectively. Then the transfection proceeded by an electric transducer (310 μV, 1500 μF). After 24 h, the medium was replaced with a fresh medium with 5 μg/ml Blasticidin. After about one week when the monoclonal appeared, the concentration of Blasticidin was reduced to 0.5 μg/ml. The cells were cultured for about 2 weeks to obtain stably transfected cell lines.

### Cell proliferation experiment

The cells were divided into six groups [RKO-Control group (RKO-Control), RKO-pEF-Negative control group (RKO-NC), RKO- NOV knock down group (RKO-NOVkd); HT115- Control group (HT115- Control), HT115-pEF-Negative control group (HT115-NC), HT115- NOV overexpression group (HT115-NPVexp)]. All six groups were treated with 5-Fu (at the concentration of IC50) every other day. After two days, the cells were digested from the flask and the density was calculated. 200 μl of cells (3000 per well) were inoculated in a 96-well plate. After culture for two days, the cells were fixed with 4% paraformaldehyde for 30 min and washed three times with PBS. Subsequently, 100 μl Crystal Violet Stain was added to each well and left to stay for 10 min. Then, 50 μl of 10% acetic acid was added and a spectrophotometer was used to measure the absorbance at 630 nm.

### Western Blot

SDS-page was performed and the proteins were transferred into PVDF membranes by the Bio-Rad mini protein II electro-transfer system. Subsequently, the proteins were blocked in 5% non-fat dried milk for 1 h at room temperature. Primary antibodies diluted in TBST were added and incubated overnight at 4 °C. After washing three times, secondary antibodies diluted in TBST (1:3000) were added and incubated for 1 h at room temperature, followed by washing three times. Finally, the proteins were photographed by chemiluminescence color rendering. The experiments were repeated three times.

### The tumor cell xenotransplantation nude mouse model

Six-week-old BALB/c nude mice were randomly divided into 6 groups [RKO-Control group (RKO-Control), RKO-pEF-Negative control group (RKO-NC), RKO- NOV knock down group (RKO-NOVkd); HT115- Control group (HT115- Control), HT115-pEF-Negative control group (HT115-NC), HT115- NOV overexpression group (HT115-NOVexp), n = 5/group]. The cell suspensions of CRC cells stably transfected with RKO-NOVkd, RKO-pEF, HT115-NOVexp, and HT115-pEF in the logarithmic growth phase were collected and inoculated into the left lateral skin of the nude mice to establish the nude mouse model. One week later, the nude mice were treated with 5-Fu (50 μmol/L, 20 mg/kg). Subsequently, the body weight and tumor volume of the nude mice were monitored after inoculation for 8 weeks. The tumor volume was calculated according to the formula: V = 0.5 × a × b^2^ (V represents the tumor volume, a = the long diameter, and b = the short diameter). At the 8th week, the nude mice were killed by cervical dislocation. The tumor was completely dissected, and the tumor volumes and masses were measured. Western blot was used to detect the relative protein expression of NOV, JNK, activator protein (AP)-1, Caspase-8, and Caspase-3 in CRC transplanted tumor tissues.

### Statistical analysis

SPSS 22.0 software (IBM, USA) was used for data analysis. Normally distributed measurement data were expressed as mean ± standard deviation (SD). The comparisons were examined by a Student-t test between two groups or ANOVA (Analysis of Variance) between multiple groups. A p-value < 0.05 was considered statistically significant.

## Results

### NOV improved the inhibitory effect of 5-Fu on CRC cells in vitro

**Fig. 1:**
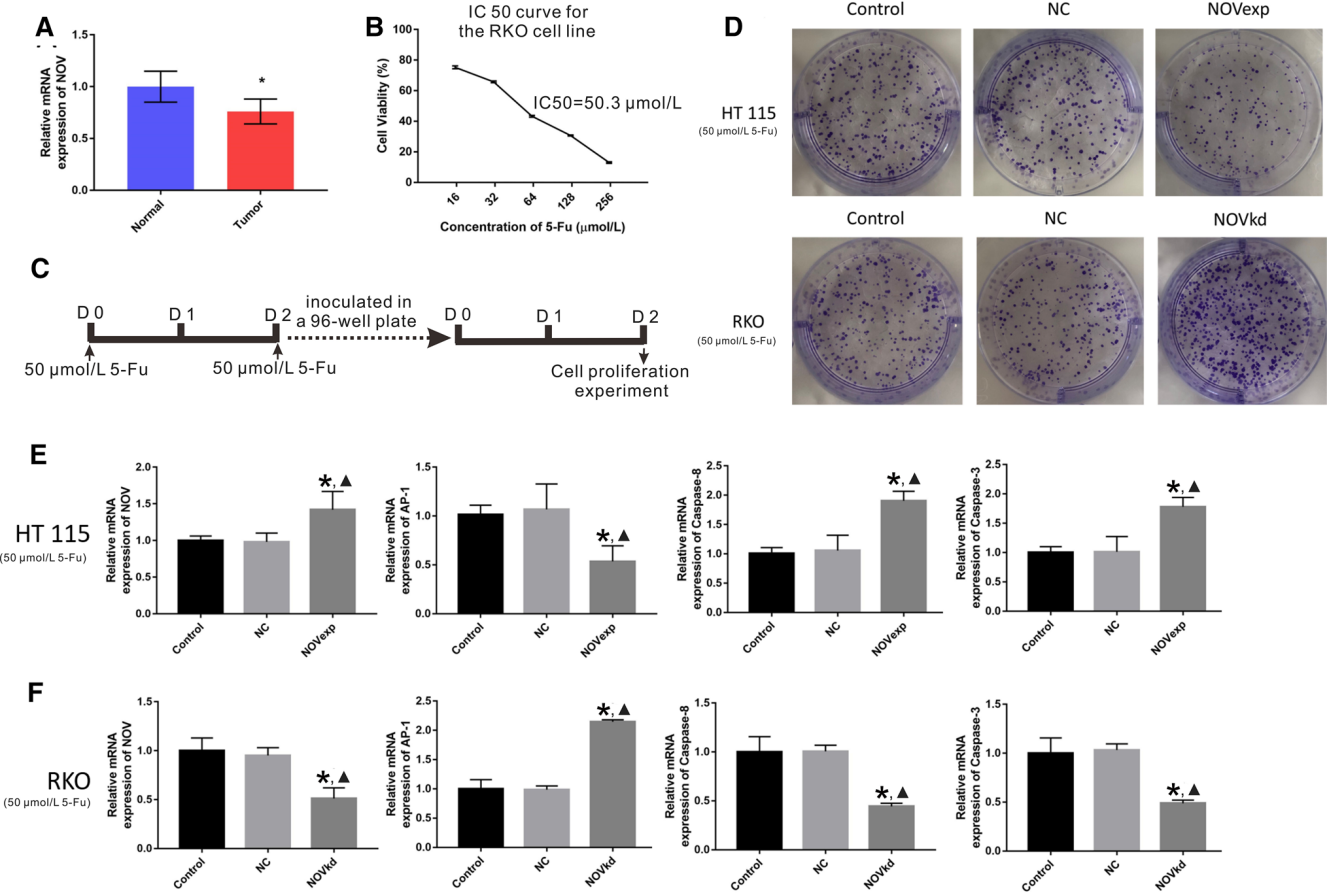
NOV improves the inhibitory effect of 5-Fu on CRC cells. **a**Examination of NOV mRNA expression in CRC and adjacent normal tissues. Ten CRC tumor specimens and adjacent normal colorectal tissue were collected during surgery. Data are shown as Mean ± SD. * P < 0.05. **b** The IC50 curve for the RKO cell line. The IC50 was 50.3 μmol/L after calculation. **c** The timeline of the cell proliferation experiment. **d** The cell proliferation assay of CRC cells. The HT115 and RKO cells were treated with each plasmid and 5-Fu with the concentration of IC50. NC: negative control, NOVexp: NOV over expression, NOVkd: NOV knock down. The mRNA expression of NOV, AP-1, Caspase-8, and Caspase-3 in HT115 (**e**) and RKO (**f**) cells by RT-PCR examination. NC: negative control, NOVexp: NOV over expression, NOVkd: NOV knock down. All groups were treated with 5-Fu (50 μmol/L). The experiments were repeated three times. Data are shown as Mean ± SD. * P < 0.05 *vs.* the control group; ▲ P < 0.05 *vs.* the NC group

**Fig. 2:**
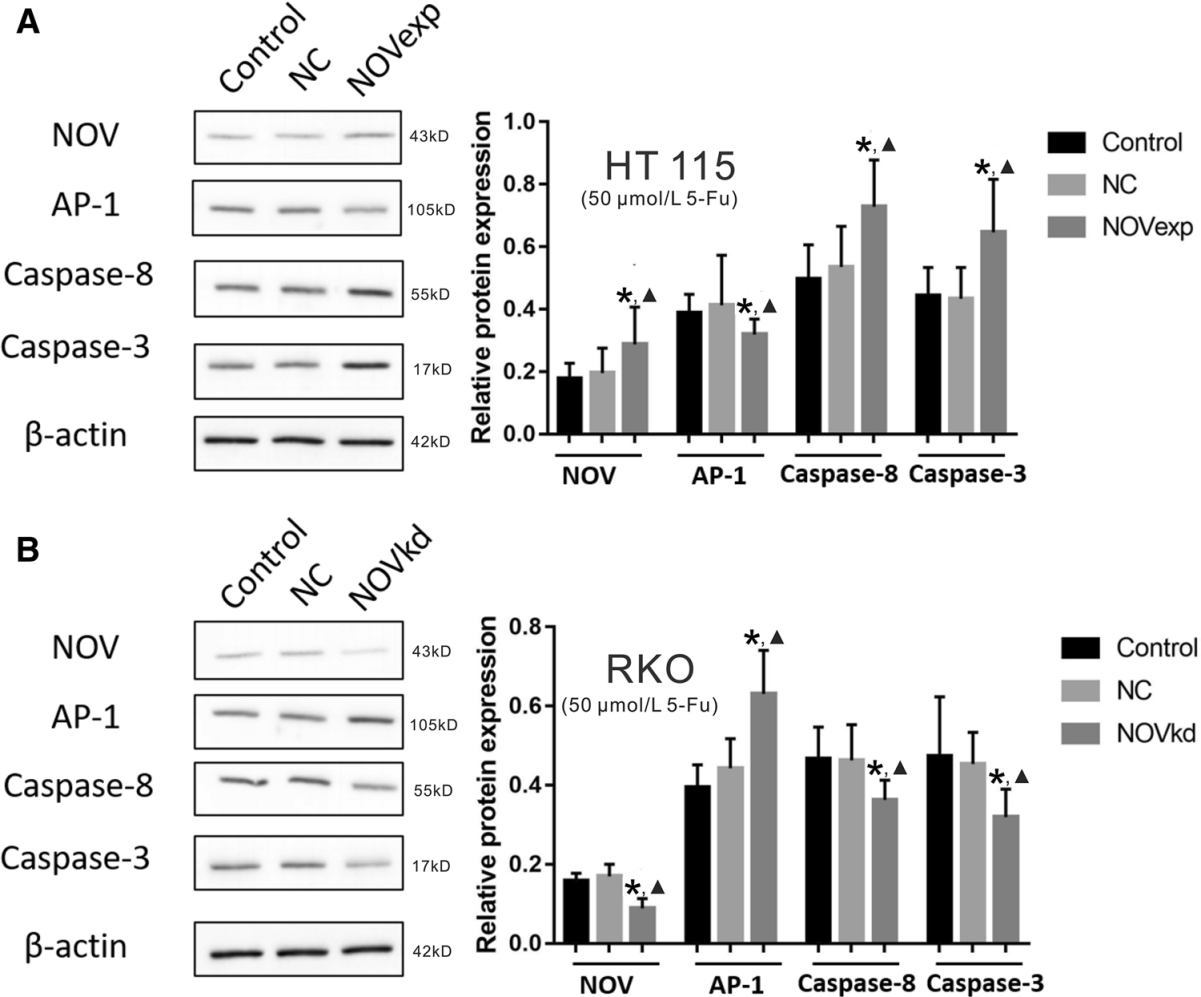
The relative protein expression of NOV, AP-1, Caspase-8, and Caspase-3 in HT115 (**a**) and RKO (**b**) cells by Western blot examination. NC: negative control, NOVexp: NOV over expression, NOVkd: NOV knock down. The cells in all groups were treated with 5-Fu (50 μmol/L). The experiments were repeated three times. Data are shown as Mean ± SD. * P < 0.05 *vs.* the control group; ▲ P < 0.05 *vs.* the NC group

We examined the NOV mRNA expression in CRC and adjacent normal tissues and found that NOV mRNA expression was significantly lower in tumor tissues than that in the normal tissues (P < 0.05) (Fig. 1a). Besides, the inhibitory effect of 5-Fu on CRC cells (HT115 cell line) was examined and the IC50 was 50.3 μmol/L after calculation (Fig. 2b). The results of the cell proliferation assay showed that there were no significant differences between the control groups and the NC groups in cancer cells in cell proliferation (Fig. 1c). However, the cell proliferation was significantly reduced in the HT115-NOVexp groups (P < 0.05) and increased in the NOVkd groups (P < 0.05) than that in the control groups and NC groups (Fig. 1c).

### NOV inhibited the expression of AP-1 and promoted the expression of Caspase-8/3 in CRC cells in vitro

Based on the observed change of cell proliferation, we further examined the potential mechanisms of NOV sensitizing CRC cells to 5-Fu treatment. First, the CRC cells with overexpression and knockout of NOV were constructed. Through examination of mRNA expression in HT115 (Fig. 1e) and RKO cells (Fig. 1f), we found that the expression of AP-1 in the HT115-NOVexp group was significantly lower (P < 0.05) than that in the HT115-NC and- control group and the expression of AP-1in the RKO-NOVkd group was significantly higher (P < 0.05) than that in the RKO-control and NC groups. Conversely, the expression of Caspase-8 and Caspase-3 was significantly higher (P < 0.05) in the HT115-NOVexp group and was significantly lower in the RKO-NOVkd group (P < 0.05) than that in the control and NC groups. The western blot results showed that the relative protein expression of AP-1, Caspase-8, and Caspase-3 was consistent with the mRNA expression (Fig. 2).

### NOV improved the inhibitory effect of 5-Fu in tumor cell xenotransplantation nude mouse model

**Fig. 3:**
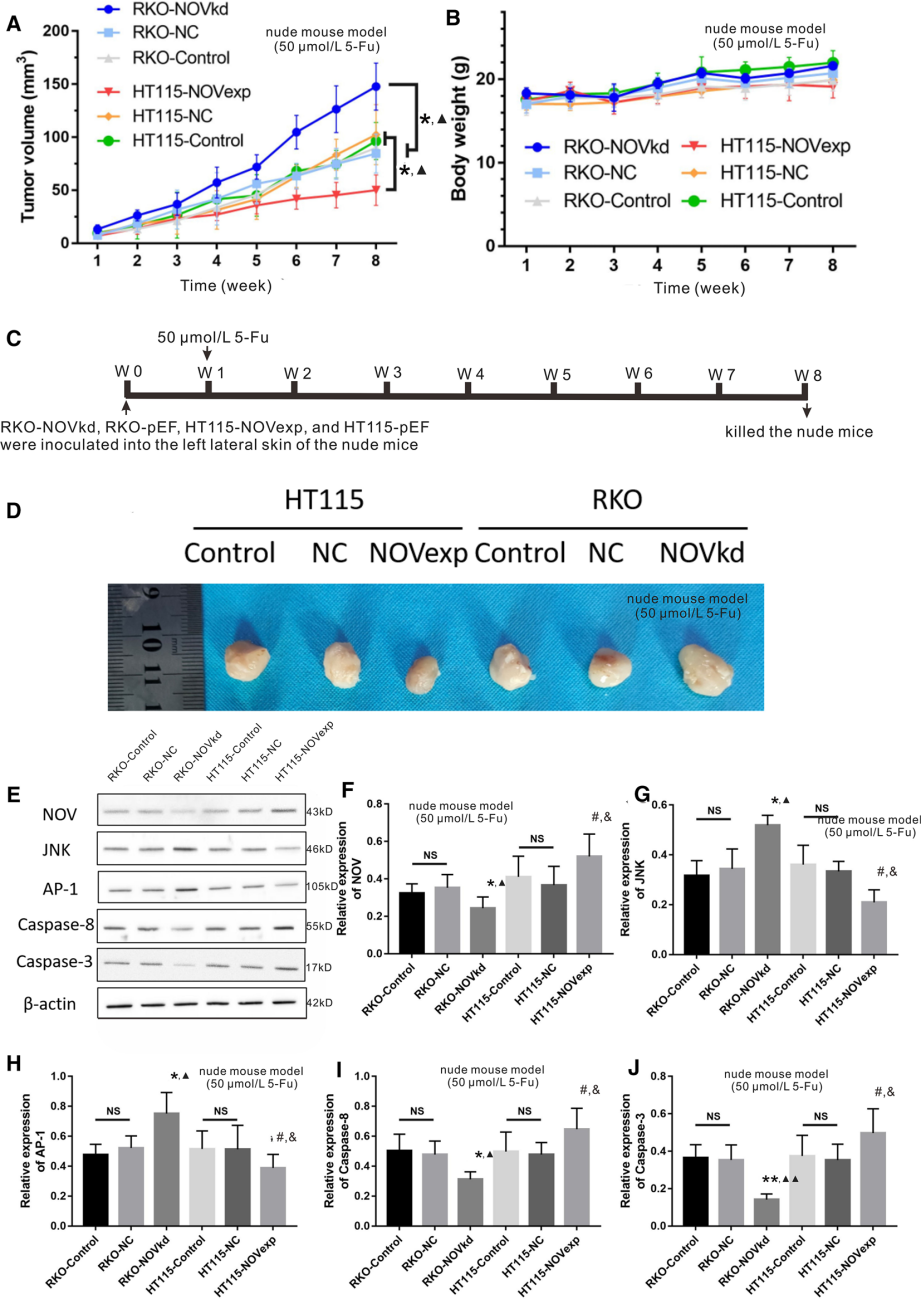
In vivo tumor cell xenotransplantation nude mouse model. The cell suspensions of CRC cells stably transfected with RKO-NOVkd, RKO-pEF, HT115-NOVexp and HT115-pEF were inoculated in the left lateral skin of nude mice (N = 5 per group). One week later, the nude mice were treated with 5-Fu (50 μmol/L). **a** Tumor volume of six groups within 8 weeks. **b** Body weight of six groups within 8 weeks. **c** The timeline of the nude mice experiment. **d** Representative photo of the tumor collected at week 8. NC: negative control, NOVexp: NOV over expression, NOVkd: NOV knock down. Data are shown as Mean ± SD. * P < 0.05 *vs.* the control group; ▲ P < 0.05 *vs.* the NC group. The relative protein expression of NOV (**f**), JNK (**g**), AP-1 (**h**), Caspase-8 (**i**), and Caspase-3 (**j**) in tumors by Western blot examination. NC: negative control, NOVexp: NOV over expression, NOVkd: NOV knock down. Data are shown as Mean ± SD. NS: no significance, * P < 0.05 *vs.* the RKO-control; ▲ P < 0.05 *vs.* the RKO-NC group; ** P < 0.01 *vs.* the RKO-control; ▲▲ P < 0.01 *vs.* the RKO-NC group; # P < 0.05 *vs.* the HT115-control; & P < 0.05 *vs.* the HT115-NC groups

To further confirm the results from in vitro experiments, an in vivo tumor cell xenotransplantation nude mouse model was established. As shown in Fig. 3a, the tumor volume in the RKO-NOVkd group was higher than that in the RKO-Control and RKO-NC groups (P < 0.05). Concurrently, the tumor volume in the HT115-NOVexp group was lower than that in the HT115-Control and HT115-NC groups (P < 0.05) (Fig. 3a). No obvious differences in body weight were observed in all the groups (P > 0.05) (Fig. 3b). The representative tumor specimens in different groups collected at week 8 were shown in Fig. 3c.

### NOV inhibited the expression of AP-1 and JUK and promoted the expression of Caspase-8/3 in vivo

The relative protein expression of NOV, JNK, AP-1, Caspase 8, and Caspase 3 in tumor tissues in tumor cell xenotransplantation nude mouse model was examined by western blot (Fig. 3d). The results showed that the relative protein expression of NOV, Caspase 8, and Caspase 3 in the HT115-NOVexp group was lower than that in the HT115-Control and HT115-NC groups (P < 0.05) (Fig. 3e, i, j), while the relative protein expression of JNK and AP-1 in the HT115-NOVexp group was higher than that in the HT115-Control and HT115-NC groups (P < 0.05) (Fig. 3g, h). Meanwhile, the relative protein expression of NOV, Caspase 8, and Caspase 3 in the RKO-NOVkd group was higher than that in the RKO-Control and RKO-NC groups (P < 0.05) (Fig. 3e, i, j), and the relative protein expression of JNK and AP-1 in the RKO-NOVkd group was higher than that in the RKO-Control and RKO-NC groups (P < 0.05) (Fig. 3g, h).

## Discussion

5-Fu will be converted into FdUMP, FdUTP, and FUMP after entering the body [18]. These three metabolite of 5-Fu can interfere with the syntheses of DNA and RNA, therefore inhibiting the proliferation of tumor cells and promoting cancer cell apoptosis [18]. 5-Fu has been regarded as the first-line drug for the treatment of advanced CRC for a long time [19]. The unsatisfactory clinical response rate of 5-Fu treatment motivated us to study the possible approach to improve its sensitization.

**Fig. 4:**
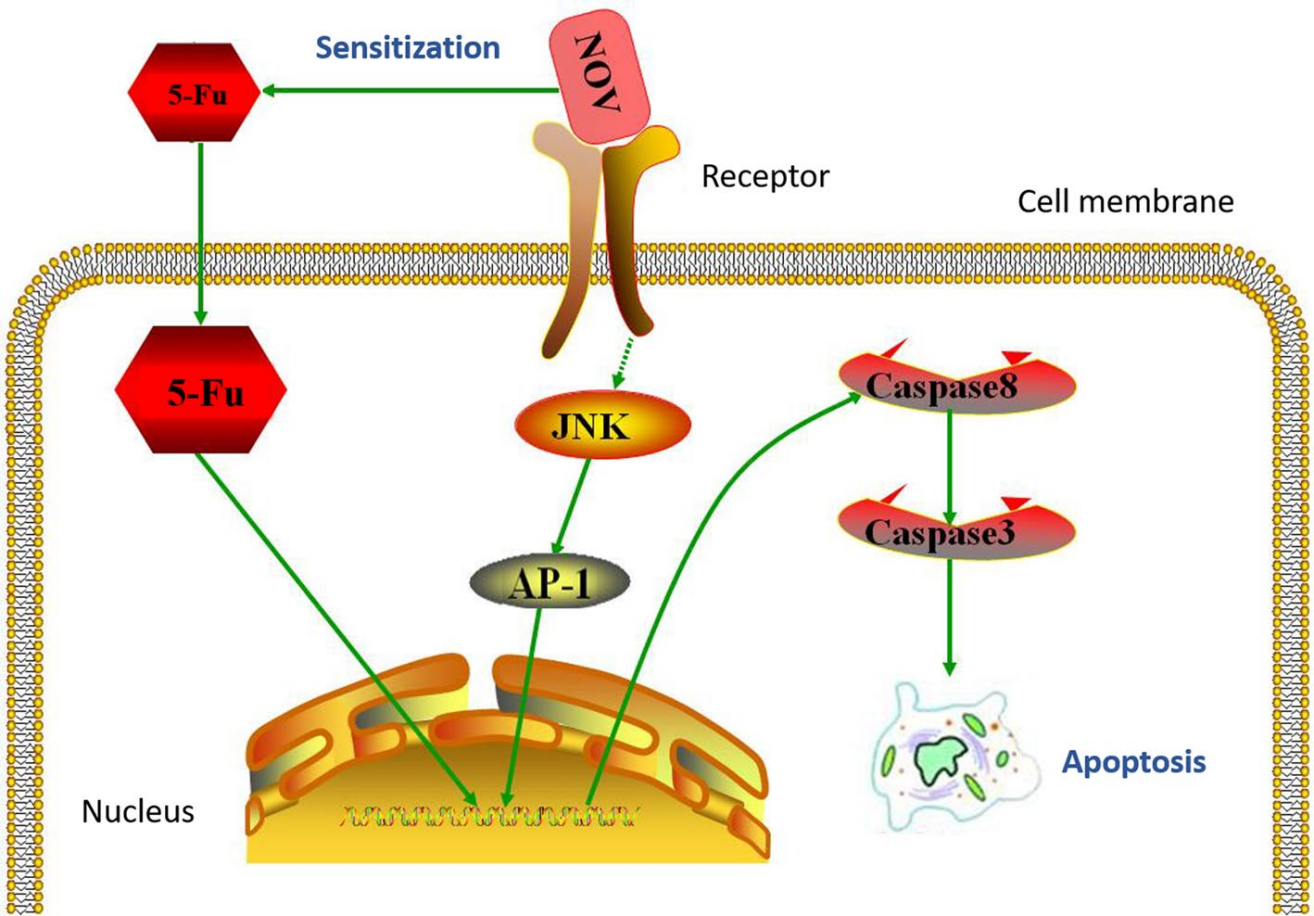
Scheme of NOV sensitizing CRC cells towards 5-Fu-mediated inhibitory effect on CRC cell proliferation through the JNK/AP-1/Caspase-8/Caspase-3 pathway

There are four conserved domains with different spatial structures from N-terminal to C-terminal of NOV, including IGFBP, VWC, TSP-1, and CT domains, which bind to different receptors [10]. NOV plays important biological functions in regulating apoptosis, angiogenesis, tumor invasion, and metastasis [20, 21]. Previous studies have reported that NOV was associated with tumorigenesis in different types of cancers such as prostate cancer [22], primary musculoskeletal tumors [23], renal cell carcinoma [24], cervical cancer [25], Ewing’s sarcoma [26], melanoma [27], and others. However, the role of NOV in colorectal cancers has been rarely reported. Through a meta-analysis, Ueda et al. found that NOV might be an indicator for poor prognosis and might be a therapeutic target in CRC [28]. Our previous study reported that NOV was possibly associated with the survival, invasion, and chemoresistance of CRC cells [17]. In this study, we first confirmed that NOV improves the inhibitory effect of 5-Fu on CRC cells in vitro and in vivo (Fig. 4).

The potential signaling pathway of NOV sensitizing CRC cells to the treatment of 5-Fu was also studied. Our results showed that knocking down the expression of NOV could improve the expression of JNK and AP-1 but decrease the expression of Caspase-8 and Caspase-3. Combined with our previous findings that the effects of NOV on the inhibition of CRC cells proliferation was mediated by a regulation of Caspase-3/-8 via JNK [17], both in vitro and in vivo results in this study suggested that JNK/AP-1/Caspase-8/Caspase-3 was a possible signaling pathway involved in the sensitization of NOV on 5-Fu in treating CRC. Zugowski et al. believed that AP-1 was related to the progress of CRC [29]. Peng et al.'s [30] study identified AP-1 as a key molecule in the regulation of CRC by nasopharyngeal carcinoma associated gene 6 (NGX6). Our study also found that AP-1 played an important role in the regulation of CRC sensitivity to 5-Fu by NOV. The promotion of apoptosis by JNK relies on two main mechanisms. On one hand, activated JNK activates the pro-apoptotic protein Bax, which is transferred from the cytoplasm to the mitochondria so that the permeability transition pores of the mitochondrial membrane are over-opened [31]. Subsequently, the release of pro-apoptotic factors such as Cyt-C, Smac, etc. into the cytoplasm activates Caspase-9, which in turn activates Caspase-3/6 to promote apoptosis. On the other hand, activated JNK enhances the activity of the transcription factor AP-1, which in turn enhances the activity of pro-apoptotic factors such as nuclear Elk-1, c-Jun, c-fos, and p53 [18, 32]. These factors promote the expression of pro-apoptotic proteins such as p53, Bax, FasL, and TNF, which would activate Caspase-8 and then Caspase-3 to promote apoptosis. Our results indicated that NOV may sensitize the inhibitory effect of 5-Fu on colorectal cancer via the JNK/AP-1/Caspase-8/Caspase-3 pathway.

In summary, NOV can sensitize CRC cells towards 5-Fu-mediated inhibitory effect on cell proliferation and this sensitization may be achieved by the JNK/AP-1/Caspase-8/Caspase-3 pathway. And our study provides an experimental basis for the potential use of NOV as a target for the promotion of 5-Fu treatment. However, drugs targeting NOV need to be explored and applied in further clinical trials to fully illuminate the actual clinical therapeutic effect. Besides, other targets associated with the chemoresistance of 5-Fu still need to be found and studied.

## Data Availability

All data generated or analysed during this study are included in this published article.
